# 379. Research nurses in Europe: education paths and key tasks, results from VACCELERATE

**DOI:** 10.1093/ofid/ofad500.449

**Published:** 2023-11-27

**Authors:** Jon Salmanton-Garcia, Fiona A Stewart, Pauline Wipfler, Sanne H I Hofstraat, Patricia Bruijning-Verhagen, Oliver A Cornely

**Affiliations:** University Hospital Cologne, Cologne, Germany, Cologne, Nordrhein-Westfalen, Germany; University Hospital Cologne, Cologne, Nordrhein-Westfalen, Germany; University Hospital Cologne, Cologne, Nordrhein-Westfalen, Germany; University Medical Centre Utrecht, Utrecht, Utrecht, Netherlands; University Medical Centre Utrecht, Utrecht, Utrecht, Netherlands; University of Cologne, Cologne, Germany, Cologne, Nordrhein-Westfalen, Germany

## Abstract

**Background:**

From January 2023, VACCELERATE offers a specific course to become “Research Nurse”. The term “Research Nurse” makes reference to nurses actively participating in the conduct of clinical trials and epidemiological studies. Despite being key to clinical research environment, the requirements of becoming a research nurse, or which tasks are assigned, might vary between countries. We aimed to depict the current situation in Europe-

**Methods:**

Relevant bodies in charge of nursing education in general, and Research Nurses in particular, were contacted, following advice of the VACCELERATE National Coordinators and the VACCELERATE Site Network. Participants were asked to complete an online survey on Research Nurse.

**Results:**

Responses from 38 countries were collected. Before becoming a Research Nurse, a nursing degree was necessary in 33 (87%) countries, for which training may take 3-5 years (Figure 1). A Good Clinical Practice (GCP) course was necessary to become a Research Nurse in 24 (63%) out of 38 countries. Overall, courses relevant for compliance with Research Nurse tasks are provided by different bodies, such as: online institutions (n=18, 47%), organisations external to the respective clinical trial unit (n=16, 42%), or on site at hospitals or universities (n=14, 37%, each). Eighteen (47%) countries reported the existence of dedicated Research Nurse courses in their boundaries, but 29 (76%) considered them necessary. Administration of investigational medicinal products (country n=33, 87%) was the most commonly assigned task, both in phase I-IV clinical trials and registry trials/observational studies. Other relevant tasks were processing of blood samples (n=32, 84%), patient training for new medicines/procedures (n=30, 79%), and shipment of clinical samples (n=29, 76%). Obtaining patient informed consent (n=18, 47%) or prescription of investigational medicinal products (n=6, 16%) was a task in only a few countries.education in their country.

**Conclusion:**

Despite the baseline educational background varying widely between countries within Europe, the tasks assigned to Research Nurses

are generally similar, which may consist of study administrative tasks, sample processing and patient training.

**Disclosures:**

**Oliver A. Cornely, MD PhD**, DZIF: Advisor/Consultant|DZIF: Board Member|DZIF: Grant/Research Support|DZIF: Honoraria|DZIF: Stocks/Bonds

